# From Unipolar to Bipolar: The Diagnostic Evolution in an Elderly Man

**DOI:** 10.1155/2023/6609793

**Published:** 2023-10-25

**Authors:** Vincent John Magat Lu, Melvyn Weibin Zhang, Giles Ming-Yee Tan, Jiangbo Ying

**Affiliations:** ^1^East Region, Institute of Mental Health, Singapore City, Singapore; ^2^Central Region, Institute of Mental Health, Singapore City, Singapore

## Abstract

A pivotal concept in the field of mood disorders is the dichotomy between unipolar depression and bipolar disorder. Due to the unique treatment in older age bipolar disorder and the scarcity of research in this area, it is clinically important to raise the awareness of the diagnostic conversion of unipolar depression to bipolar disorder in the elderly population. We present a case of a 71-year-old Chinese man whose diagnosis was revised to bipolar disorder after 9 years of treatment for unipolar depression. Organic workup, including blood tests and brain imaging, was performed to rule out organic causes. This patient eventually responded well to the combined treatment of an antipsychotic and a mood stabilizer. This case report adds to the growing literature of challenges in identifying and managing bipolar disorder in the elderly. As unipolar depression and bipolar disorder have different disease courses and different treatment strategies, it is essential for clinicians to be aware of diagnostic conversion. Further research is needed to delineate bipolar disorder from unipolar depression in the elderly population.

## 1. Introduction

A pivotal concept in the field of mood disorders is the dichotomy between unipolar depression and bipolar disorder. The categorization implies that patients are diagnosed with unipolar depression, rather than bipolar disorder, as long as they have not exhibited hypomanic or manic symptoms [1]. However, diagnostic conversion from unipolar depression to bipolar disorder is not an uncommon occurrence in the practice of psychiatry. Studies have shown that about 5%–10% of patients who initially present with unipolar depression will have a manic episode in about 6–10 years, with the mean age of switch at about 32 years [2]. The risk of conversion appears to be related to the number of depressive episodes prior to the onset of the first manic episode [3] as well as the severity of the depressive episode(s) [4].

In the elderly population, the risk of developing a first manic or hypomanic episode remains present, although relatively uncommon. Thus, there is need to have a high index of suspicion regarding other causes, such as delirium, dementia, and other nonpsychiatric medical conditions [2]. According to the International Society for Bipolar Disorders Task Force [5], older age bipolar disorder is defined as bipolar disorder in individuals aged 50 years or above, and it includes: (1) early onset bipolar disorder, in which the age at first manic or hypomanic episode is below 40 years and (2) late-onset bipolar disorder, in which the age of onset is 40 years and above. Older age bipolar disorder is associated with notable cognitive deficits [6], frequently cooccurring physical health conditions [7], and impaired psychosocial functioning [8]. Individuals who present with manic episodes late in life are reported to have significantly higher mortality rates [3]. When treating older age bipolar disorder, clinicians need to pay close attention to the side effects and comorbidities [9].

Although the elderly constitutes the most rapidly expanding segment of the population in the world, research data on older age bipolar disorder are sparse [9]. There is a need to explore in the field related to the diagnostic conversion from unipolar depression to older age bipolar disorder. There are inconsistent findings in this area. For example, it has been reported in several studies that the diagnostic conversion from unipolar depression to bipolar disorder is associated with younger age at onset, instead of elder age [10, 11]. However, a recent study has indicated that the onset of depression in late life may represent an unstable state with a significant risk of transitioning into bipolar disorder [12].

Due to the unique treatment in older age bipolar disorder and the lack of sufficient research in this area, it is clinically important to raise the awareness of the conversion of unipolar depression to bipolar disorder in the elderly population. Here, we present a case of a 71-year-old Chinese man whose diagnosis was revised to bipolar disorder after 9 years of treatment for unipolar depression. This case report adds to the growing literature of challenges in identifying and managing bipolar disorder in the elderly.

## 2. Case Presentation

A 62-year-old Chinese man first presented to our hospital in October 2014 for worsening of low mood. At that time, he had just been seen by a private psychiatrist and was diagnosed with major depressive disorder. Aside from the depressive symptoms, he also manifested with strong nihilistic delusions. He did not have any significant physical health conditions. The patient was subsequently admitted to our hospital as a case of major depressive disorder with psychotic features and treated with mirtazapine 30 mg every night, venlafaxine 150 mg every morning, and risperidone 1 mg every night. During his hospitalization, he also underwent six sessions of electroconvulsive therapy (ECT) and improved significantly after 2 months on the ward.

Upon discharge, he was maintained on mirtazapine, venlafaxine, and risperidone for 2 years. In 2016, risperidone was discontinued. In 2018, due to his stable mood, venlafaxine was also discontinued, and he was only maintained on mirtazapine 30 mg every night. Over the years, he was continued on mirtazapine with doses fluctuating between 15 and 30 mg daily depending on the stress that he experienced at that time. He did not display any manic or hypomanic symptoms during this long period when he was only on mirtazapine. He managed to resume work as a school cleaner and continued on through the years with no significant issues. He was on 6-monthly follow-ups and had been generally compliant with his antidepressant medication.

The patient was apparently well until May 2023 when he was 71 years old. He was then brought to our hospital again by his relatives for evaluation after a change in behavior for 2 weeks. The patient was observed to have significant mood swings and was described to be very active and irritable. He was also noted to have started sending messages with grandiose content and overspending his money. He was physically aggressive toward his relatives, prompting them to bring him to our psychiatric emergency room for evaluation. During the consult, the patient's mood was observed to be labile. His thought process was largely goal directed but was noted to be interspersed with tangential and at times incoherent responses. He endorsed a decreased need for sleep, racing thoughts, and grandiose delusions. The patient did not take any illicit drugs and did not have any family history of mental illness. There were also no new medications given to him recently. Due to the acute change in his mental state, he received a comprehensive organic workup where blood examination along with computed tomography and magnetic resonance imaging of the brain revealed unremarkable results.

The patient's diagnosis was revisited, and his treatment plan was revised. Mirtazapine was discontinued to reduce the risk of worsening his manic symptoms. He was started on olanzapine 20 mg daily to treat his mania. However, the patient's manic symptoms persisted, though reduced in intensity. Valproic acid 500 mg daily was then added. The manic symptoms eventually resolved with the combined treatment of olanzapine and valproic acid. The patient was finally diagnosed as suffering from bipolar 1 disorder.

## 3. Discussion

It is generally a challenge to predict whether a patient who presents with depression will eventually convert to bipolar disorder. In the case presented here, the elderly Chinese man, who had no family history of mental illness, initially presented with depressive symptoms, and then manifested manic symptoms after 9 years of treatment for unipolar depression. His diagnosis was revised to bipolar disorder when he was 71 years old.

Because the patient was on antidepressant treatment when he became manic, medication-induced bipolar disorder might be considered as one of the differential diagnoses. However, medication-induced bipolar disorder is unlikely, after the comprehensive longitudinal history was considered. The patient had been on mirtazapine for many years, and he did not have any manic or hypomanic symptoms until recently. Besides, the Diagnostic and Statistical Manual of Mental Disorders, Fifth Edition, Text Revision (DSM-5-TR) states that manic episodes that emerge in the context of treatment with antidepressant medication and persist at a fully syndromal level beyond the physiological effect of the medication warrant a diagnosis of bipolar disorder [1]. In this case report, the patient's manic symptoms persisted after mirtazapine was stopped and only resolved after the combined treatment of olanzapine and valproic acid. Due to the persisted symptoms beyond the pharmacological effect of mirtazapine, a diagnosis of primary bipolar disorder is more appropriate. Nonetheless, it is worthwhile to note that the antidepressant treatment in the elderly is associated with an increased incidence of mania/bipolar disorder ranging from 13.1 to 19.1 per 1,000 person-years, compared to the overall incidence rate of mania/bipolar disorder that is 10.9 per 1,000 person-years [13]. It is also noteworthy that in the diagnosis of bipolar disorder in the elderly, clinicians must have a high index of suspicion and attempt to rule out other medical or neurological conditions that may present with similar symptoms, as there has also been increasing evidence of late-onset mania having an underlying organic cause [14]. Besides, clinicians also need to focus on the assessment of comorbidities, the use of new medications, and underlying neurological mechanisms when diagnosing this population [15].

The risk of unipolar depression developing into bipolar disorder remains constant lifelong [10]. Among new cases diagnosed with bipolar disorder, approximately 6%–8% are older than 60 years [16]. Several risk factors related to this diagnostic conversion have been reported. Our case had no family history of mental illness and initially presented with depressive symptoms with nihilistic delusions. These clinical factors are partly consistent with the results in other studies. Family history of affective disorders increases the risk of conversion to bipolar disorder [17, 18] and the presence of psychotic depression is associated with a higher risk of bipolar disorder [19]. However, our case had some unique features, as it is a case of an elderly man who was initially treated as late-onset depression. Conversion rates are highest for female patients aged 18–29 years and gradually decreased with age until 60–69 years [20], but our patient is a man and was diagnosed with depression when 62 years old and converted to bipolar disorder at the age of 71 years. Moreover, faster conversion to bipolar disorder has been noted in patients presenting with late-onset depression, which may represent a more unstable condition compared to the general population [12], but in our patient it took 9 years before the conversion. These highlight the importance to remain vigilant when managing elderly patients with depression without typical risk factors for conversion to bipolar disorder.

With the aging population globally, there is an anticipated increase in the prevalence of bipolar disorder in the elderly. However, the number of studies that focus on older age bipolar disorder remains scarce. It is crucial to recognize distinctive characteristics that are more common in the elderly population, such as a higher prevalence of psychiatric and medical comorbidities. These factors, in turn, impact the treatment approach, as clinicians must carefully consider these comorbidities and take into account physiological differences that can influence the response to medications [21]. It has been reported that there are no dedicated sections addressing the specific elderly population in the treatment guidelines in the management of bipolar disorder, so the current management has predominantly followed approaches similar to those used for younger age groups, with a focus on taking age and any concurrent medical conditions into careful consideration [22]. The age-related changes in terms of pharmacodynamics and pharmacokinetics should also be considered when initiating medication in this population [23]. Mixed-age studies have demonstrated the efficacy of commonly used medications in the elderly population along with additional psychological treatments [24]. Preliminary studies in the treatment of the elderly population have revealed the benefits of the role of ECT and psychosocial interventions [23]. Closer follow-up in the community may also be beneficial for this population as elderly individuals have frailty, impaired functioning, and 1.5–2-fold higher mortality risk [15]. It is recommended that clinicians should approach the treatment of bipolar disorder in the elderly with an integrative care model that aims to enhance psychosocial functioning and overall quality of life [25].

Clinicians need to be aware of the risk of conversion to bipolar disorder among elderly patients with unipolar depression, due to the different pharmacological choices between the two conditions. It is crucial to exercise caution and closely monitor the prescription of antidepressants in patients with late-onset depression to prevent mood destabilization [26]. When treating late-onset depression among elderly patients, clinicians need to monitor for any manic or hypomanic symptoms. If manic or hypomanic symptoms occur, psychotropics with mood stabilizing effects, such as lithium, can be considered. Lithium has demonstrated effectiveness in preventing episodes of both recurrent depression and bipolar disorder in the elderly patients [27, 28]. However, several factors need to be considered when starting lithium in the elderly patients, including lithium toxicity [29] and renal adverse events [30]. Lithium dose reduction is frequently necessary for older patients, with suggested serum levels of 0.4–0.8 mmol/L for those aged 60–79 years and 0.4–0.7 mmol/L for those aged 80 years or older [31].

In conclusion, forecasting diagnostic conversion from depression to bipolar disorder and managing bipolar disorder in the elderly population pose significant challenges. This case report highlights the clinical significance of monitoring the conversion of unipolar depression to bipolar disorder in the elderly patients. As unipolar depression and bipolar disorder have different disease courses and different treatment strategies, it is important for clinicians to be vigilant about diagnostic conversion. Further research is needed to delineate bipolar disorder from unipolar depression in the elderly population.

## Data Availability

The data used to support the findings of this study are included within the article.
